# Society Meetings

**Published:** 1895-10

**Authors:** 


### Society Meetings.

---

Buffalo Academy of Medicine.—*Section of Medicine.*—List of papers and discussions :

September 10, 1895, discussion : Should the present law concerning instruction in the public schools upon the effects of alcohol be repealed ? Yes, Dr. H. R. Hopkins. No, Rev. Frank S. Fitch.

October 8th, papers: Gastritis, Dr. A. L. Benedict; Report of an unusual case of purpura, Dr. A. E. Diehl; Tetany, report of five cases, Dr. Wm. C. Krauss, Dr. A. H. Briggs.

November 12th, discussion: Should the Crede method of treatment of the eye in new-born children be carried out in daily practice? Yes, Dr. A. A. Hubbell, Dr. A. G. Bennett. No, Dr. R. L. Banta, Dr. Sidney A. Dunham.

December 10th, Medical Section entertains the Academy.

January 14, 1896, papers: Some of the more important relations of the eye to general practice, Dr. B. H. Grove; Painful urination, Dr. W. H. Heath; Affections of the lingual tonsils, Dr. W. S. Renner.

February 11th, discussion: What are the best methods of medical teaching? Discussion opened by Dr. Henry C. Buswell; A plea for the apprenticeship, Dr. P. W. Van Peyma; For more Hospital Work, Dr. Chas. Cary; For more laboratory work, Dr. Roswell Park.

March 10th, papers: Dilatation of the stomach, Dr. Allen A. Jones; Forcible respiration and pneumatic cabinet, Dr. J. H. Pryor; On the value of forced respiration in saving human life in chloroform, ether and nitrous oxide narcosis, Dr. George E. Fell.

April 14th, discussion: Is creosote of advantage in tuberculosis? Yes, Dr. DeLancey Rochester. No, Dr. J. H. Pryor.

May 12th, papers: Membranous enteritis, Dr. Lillian C. Randall; Cystitis, Dr. W. D. Greene; Intestinal stone, with specimen, Dr. W. C. Phelps.

June 9th, discussion: Have medical men a moral right to patent medical instruments? Yes, Dr. George E. Fell. No, Dr. Herman E. Hayd.

Meetings will commence promptly at 8.45 P. M. Papers should not exceed fifteen minutes, nor all the discussions on a paper ten minutes. Preference given to those who bring patients for demonstration.

DR. LUCIEN HOWE, *President.*

DR. IRVING W. POTTER, *Secretary.*

Advisory committee, Dr. Charles G. Stockton, Dr. Henry C. Buswell, Dr. H. R. Hopkins.

---

THE Mississippi Valley Medical Association, at its annual meeting, held in Detroit, September 3–6, 1895, elected the following officers: President, Dr. H. O. Walker, of Detroit; vice-presidents, Dr. B. Merill Rickets, of Cincinnati, and Dr. Frederick C. Woodburn, of

Indianapolis; secretary, Dr. H. W. Loeb, of St. Louis; treasurer, Dr. Harold N. Moyer, of Chicago. The next annual meeting will be held in St. Paul, Minn.

---

THE Tri-State Medical Society, of Alabama, Georgia and Tennessee, will hold its next annual meeting at Chattanooga, Tuesday, Wednesday and Thursday, October 8, 9 and 10, 1895. Dr. Frank Trester Smith, the secretary, has issued a preliminary program, which bespeaks an interesting meeting.

---

THE MEDICAL Association of Central New York will hold its twenty-eighth annual meeting in Syracuse, Tuesday, October 15, 1895, under the presidency of Dr. Floyd S. Crego, of Buffalo. Dr. E. B. Angell, of Rochester, the secretary, is preparing a program for the occasion.

---

THE American Public Health Association holds its twenty-third annual meeting at Denver, Tuesday, Wednesday, Thursday and Friday, October 1, 2, 3 and 4, 1895, under the presidency of Dr. William Bailey, of Louisville. Dr. Irving A. Watson, of Concord, N. H., is the secretary.

